# Chromolithographic Eyeground

**Published:** 1864-02

**Authors:** 


Chromolithographic Eyeground.—The next number of the American Journal of Ophthalmology is to contain a chromolithographic view of the eyeground. We have received a specimen of the representation, and regard it as very beautiful. Those who are paying attention to the diseases of the eye, will be greatly interested in it. We have no doubt the representation is truthful and correct, though we are frank to confess that the vessels, optic nerve, etc., are better defined in the picture than we have ever been able to obtain them in the living subject. Hope we may see clearer hereafter.

